# No Indices of Increased Type 2 Diabetes Risk in Individuals with Reactive Postprandial Hypoglycemia

**DOI:** 10.3390/metabo12121232

**Published:** 2022-12-08

**Authors:** Špela Volčanšek, Urška Rahne Perc, Mojca Lunder, Draženka Pongrac Barlovič

**Affiliations:** 1 Clinical Department of Endocrinology, Diabetes and Metabolic Diseases, University Medical Centre Ljubljana, Zaloška cesta 7, SI-1000 Ljubljana, Slovenia; 2 Medical Faculty Ljubljana, University of Ljubljana, Vrazov trg 2, SI-1000 Ljubljana, Slovenia

**Keywords:** oral glucose tolerance test, diabetes screening, insulin resistance, insulin sensitivity, beta-cell function, reactive postprandial hypoglycemia

## Abstract

Reactive postprandial hypoglycemia (RPH) is an understudied condition that lacks clinical definition, knowledge of future health implications, and an understanding of precise underlying mechanisms. Therefore, our study aimed to assess the glycemic response after glucose ingestion in individuals several years after the initial evaluation of RPH and to compare glucose regulation in individuals with RPH vs. healthy volunteers. We assessed the inter- and intra-individual differences in glucose, insulin, and C-peptide concentrations during 5-h oral glucose tolerance tests (OGTTs); the surrogate markers of insulin resistance (HOMA-IR and Matsuda index); and beta-cell function (distribution index and insulinogenic index). The study included 29 subjects with RPH (all females, aged 39 (28, 46) years) and 11 sex-, age-, and body mass index (BMI)-matched controls. No biochemical deterioration of beta-cell secretory capacity and no progression to dysglycemia after 6.4 ± 4.2 years of follow-up were detected. RPH subjects were not insulin resistant, and their insulin sensitivity did not deteriorate. RPH subjects exhibited no differences in concentrations or in the shape of the glucose-insulin curves during the 5-h OGTTs compared to age- and BMI-matched controls. No increased incident type 2 diabetes risk indices were evident in individuals with RPH. This dictates the need for further research to investigate the magnitude of future diabetes risk in individuals experiencing RPH.

## 1. Introduction

The most common cause of non-diabetic hypoglycemia in a seemingly healthy individual is reactive hypoglycemia, also referred to as reactive postprandial hypoglycemia (RPH), idiopathic reactive hypoglycemia, or postprandial hyperinsulinemic hypoglycemia syndrome [1,2,3]. RPH is characterized by adrenergic and neuroglycopenic symptoms occurring 2–5 h following carbohydrate ingestion [2,4]. The future health implications and prevalence of RPH in the general population are rarely estimated [5]; rare reports state that it is experienced by up to 40% of women [6,7]. Symptoms, associated with RPH, can be effectively reduced by interventions focused on healthy modifications in dietary habits that may reduce the severity of postprandial symptoms attributed to hypoglycemia [8]. Nevertheless, RPH is a common cause for referral to an endocrinologist where an extensive diagnostic evaluation is frequently needed to exclude an organic cause of hypoglycemia [1,4,9,10]. 

The underlying mechanisms of hypoglycemia in the absence of diabetes remain controversial and employ aberrations in the hormonal milieu ranging from the entero-insular axis to renal glucosuria [2,11]. However, it is most commonly hypothesized that RPH occurs due to an excessive or delayed insulin response to a glucose load [12,13] and altered insulin sensitivity [14]; it could, therefore, represent one of the first manifestations of a glucose-regulation defect. Consequently, RPH is historically associated with an increased risk of later type 2 diabetes development [2,3,14]. In addition, subjects who experience hypoglycemia frequently engage in obesogenic behaviors to avoid symptoms, which could also add to the increased type 2 diabetes risk [6]. Thus, it is advantageous to identify metabolic dysfunction before type 2 diabetes onset in order to implement preventative strategies, diabetes screening [15] and to offer RPH-experiencing subjects a piece of reliable lifestyle advice. 

Therefore, we aimed to understand more in detail the glycemic response after glucose ingestion and to exploit the information obtained with a 5-h OGTT to estimate insulin sensitivity and beta-cell function in individuals with RPH. In addition, we compared the glycemic response of individuals several years after the initial evaluation of RPH and sought to assess the risk for possible new-onset type 2 diabetes cases. Furthermore, we compared the glycemic response to the glucose load of individuals with RPH to healthy volunteers. 

## 2. Methods

**Scheme 1:**
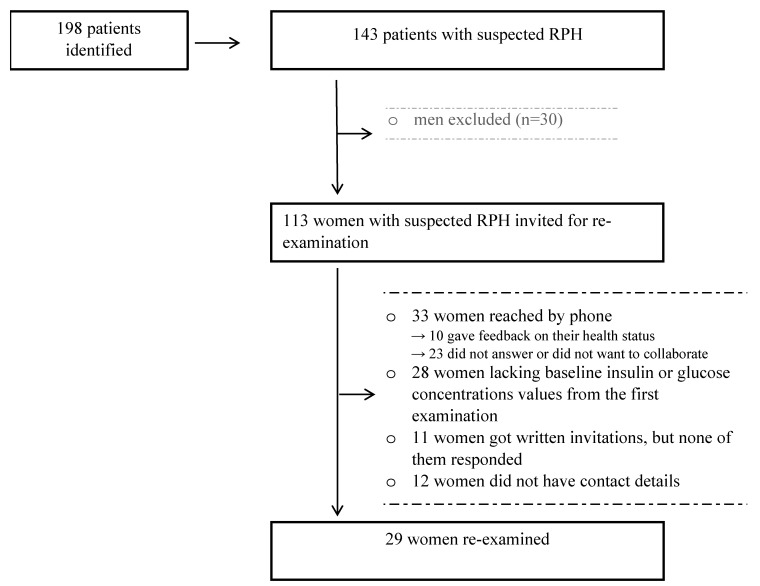
Design of the study. Legend: RPH: reactive postprandial hypoglycemia.

The present study had a prospective cohort design. The medical files of all the individuals that were referred to the outpatient clinic of Department of Endocrinology, Diabetes and Metabolic Diseases, University Medical Centre Ljubljana, under the suspicion of non-diabetic hypoglycemia from January 1994 to December 2017 (*n* = 198) were reviewed (Scheme 1). Subjects who were diagnosed with RPH after evaluation at the outpatient clinic in the described time window were invited for a follow-up examination to redefine glycemic response and identify possible new-onset type 2 diabetes cases. To ensure a greater homogeneity of the group, we invited only females, who predominated among individuals with RPH (80%). The exclusion criteria were impaired fasting glucose (IFG) and impaired glucose tolerance (IGT) at the time of presentation, pregnancy, history of any gastrointestinal surgery that induces malabsorption, and history of other identified conditions that cause hypoglycemia (malignancy, infection, adrenal insufficiency, and untreated thyroid disease). Reasons for the non-inclusion of the remaining patients were the following: refusal to participate, lack of contact information, lack of response to a written invitation, and lack of baseline insulin or glucose concentrations during the OGTT (Scheme 1).

In parallel, a group of healthy individuals without a history of RPH was invited to participate in the study, considering the basic characteristics of the examinees (female gender, average age, BMI, and lack of significantly associated diseases). The control group of healthy individuals was composed of staff, relatives, and acquaintances of healthcare workers at the clinical department.

Ethical approval was obtained from the Slovenian National Ethical Committee (No. 0120–706/2017/4.3). All the subjects were informed of the aims of the study, which was conducted following the Declaration of Helsinki. Written informed consent was obtained from each included individual before entering the study.

### 2.1. Definition of Clinical and Laboratory Characteristics

In the prospective part of the study, a detailed personal history was taken, and a clinical examination was performed, including basic anthropometric measurements, such as body weight, height, body mass index (BMI), waist and hip circumference, blood pressure, and heart rate.

A spectrum of laboratory analyses was performed, including a 5-h OGTT with 75 g of glucose and the determination of serum glucose (mmol/L), insulin, and C-peptide after 0, 30, 60, 120, 180, 240, and 300 min. Blood glucose concentration was analyzed with an automatic Avria LabCell-Siemens system. Insulin and C-peptide were assayed using an enzyme-labeled chemiluminescent immunometric assay (Siemens Healthcare Diagnostics, Deerfield, IL, USA) and were expressed in mU/L and nmol/L, respectively. The following were the references laboratory values: serum insulin concentration (fasting state) of 2–17.2 mU/L; serum C-peptide concentration (fasting state) of 0.3–2.4 nmol/L. Glycated hemoglobin was measured using high-performance liquid chromatography with a BioRad D-100 automatic analyzer. Hypoglycemia was defined as a venous-blood glucose concentration of 3.5 mmol/L or lower, accompanied by the signs and symptoms of hypoglycemia and the following resolution after the ingestion of carbohydrates (Whipple triad).

Five-hour OGTTs

The participants were advised to comply with three days of unlimited carbohydrate intake before the OGTT. The test was performed between 7 and 9 a.m., following 10 h of fasting. The subjects ingested 75 g of glucose dissolved in 250 to 300 mL of water within 5 min. The first blood sample was taken before the ingestion of glucose solution (in 0. min) and then at the 30th, 60th, 120th, 180th, 240th, and 300th minute. In the event of hypoglycemia occurrence during the test, the test was terminated prematurely, and a blood sample was taken again to determine serum glucose, insulin, and C-peptide concentrations. 

Various indices of insulin sensitivity, insulin resistance, and beta-cell secretory function were calculated using the data from the OGTTs.

Homeostatic Model Assessment for Insulin Resistance (HOMA-IR) [16] is a widely used index for assessing insulin resistance. The index assumes a constant balance between beta-cell insulin and steady-state hepatic glucose release. The normal range of values is less than 2.5. Fasting glucose and insulin concentrations were used for the calculation of the following equation:

HOMA-IR = (glucose concentration in 0. min (mmol/L) · insulin concentration in 0. min (mU/L))/22.5

The Matsuda index [17] is an insulin resistance index that represents both hepatic insulin resistance and peripheral tissue resistance to insulin. Values greater than 4.3 represent normal whole-body insulin sensitivity. The following equation was used for the calculation:

MATSUDA INDEX = (10 000)/√ ((glucose concentration concentration in 0. min (mmol/L) × insulin concentration in 0. min (mU/L) × average glucose concentration during 5-h OGTT test (mmol/L) × average insulin concentration during 5-h OGTT test (mU/L)))

Insulin secretion and thus the function of beta cells can be assessed using the disposition index and the insulinogenic index (IGI index) [18,19]. The equations for calculating both indices are the following:

INSULINOGENIC INDEX (IGI INDEX) = (insulin concentration in 30th min (mU/L) − insulin concentration in 0. min (mU/L))//((glucose concentration in 30th min (mmol/L) − glucose concentration in 0. min (mmol/L)))(1)

Defect in insulin secretion if < 0.4 [20].

DISPOSITION INDEX = IGI/(HOMA-IR)(2)

Normal range > 1 [18,19].

### 2.2. Statistical Analysis

Variables were checked for normal distribution using the Kolmogorov–Smirnov test. The normally distributed variables were reported as averages ± standard deviation (SD). Non-normally distributed variables were reported as medians (25th and 75th percentiles). To analyze the differences in individuals who were invited to a repeated 5-h OGTT and to assess possible changes in the pattern of insulin secretion and blood glucose concentrations, we used Student’s paired *t*-test. We used the Mann–Whitney U test or the chi-square test for non-normally distributed variables. A *p*-value of less than 0.05 was determined as the limit of statistical significance. Due to the preliminary nature of the study, it was not possible to calculate the sample size needed to obtain a power greater than 80% with an α error of less than 5%. Data were reported and analyzed using the statistical computer program SPSS 23 (Statistical Package for Social Sciences; version 23.0; SPSS Inc., Chicago, IL, USA).

## 3. Results

### 3.1. Characteristics of the Cohort of Individuals with RPH

Initially, 113 female patients were diagnosed with RPH from January 1994 to December 2017 at the University Medical Centre Ljubljana outpatient clinic. The most prevalent symptoms at their presentation were: trembling (41%), sweating (37%), weakness (28%), nausea (28%), hunger (17%), vertigo (17%), fatigue (11%), blurred vision (8%), palpitations (6%), and/or headache (4%). The study population was exclusively female, with median age of 39 (28, 46) years, who were re-evaluated 6.4 ± 4.2 years after their initial visit to the clinic. At the time of follow-up, the following values were recorded: mean systolic blood pressure of 107 ± 10 mmHg and diastolic blood pressure of 70 ± 7 mmHg, and waist circumference of 84.9 ± 12.7 cm and hip circumference of 102.8 ± 9.0 cm, which added up to a waist-to-hip ratio (WHR) of 0.82 ± 0.11. Their BMI was numerically higher, but the difference was not statistically significant. However, HbA1c was significantly lower at the follow-up visit. No significant differences in the indices of insulin sensitivity or resistance were noted. Detailed relevant characteristics of the study population are presented in Table 1.

Positive family history of diabetes was present in 48%. Of the concomitant diseases, the most common were gastroesophageal reflux disease (17%), mental disorders (depression, 17%), thyroid disease (13%), polycystic ovary syndrome (PCOS) (10%), and asthma (7%). Pharmacotherapy of comorbidities in RPH participants is listed by frequency in Supplementary Table S1.

### 3.2. Glycemic Response upon Glucose Stimulation in Individuals with RPH at Baseline and Follow-Up

In the OGTTs, peak glucose concentration was reached in the 30th minute (7.2 ± 1.5 mmol/L), and peak insulin concentration in the 60th minute (64.6 ± 30.5 mU/L) of the OGTT. Hypoglycemia occurred most frequently in the 180th minute or 240th minute and not before the 120th minute. The glucose concentrations at the 240th and 300th minutes were significantly lower at follow-up. The concentrations of blood glucose, insulin, and C-peptide at different time points during the OGTTs are presented in Supplementary Table S2.

### 3.3. Presentation of Symptoms in Subjects with RPH

At the first visit the symptoms reported during RPH were the following: weakness (60.9%), tremors (48.6%), nausea (37.5%), hunger (34.8%), sweating (29.4%), and dizziness (29.2%).

At follow-up, 21 subjects (72,4%) still reported the occurrence of hypoglycemic symptoms, while the remaining 8 individuals no longer experienced any symptoms of hypoglycemia. The following symptoms were those reported most often with hypoglycemia occurrence: weakness (51.3%), tremors (62.5%), nausea (15.8%), hunger (35.0%), sweating (42.5%), and dizziness (35.0%).

### 3.4. New-Onset Diabetes Occurrence 

In the 6.4 ± 4.2 years between the first OGTT and the second OGTT, none of the subjects in the prospective part of the study developed type 2 diabetes.

### 3.5. Characteristics of Subjects with the RPH and Control Group

The control group comprised age-, BMI-, WHR-, blood-pressure-, and HbA1c-matched healthy subjects (*n* = 11), all females. No significant differences in anthropometrics or indices of insulin sensitivity or resistance were evident in comparison with the RPH subjects, as shown in Table 2. The only significant difference was a lower insulinogenic index in subjects with RPH (*p* = 0.028), indicating blunted first-phase insulin response; however, it did not reach the threshold defining a defect in insulin secretion (cut-off point of 0.4). 

### 3.6. Comparison of Biochemical Characteristics of Subjects with RPH vs. the Control Group

**Figure 1:**
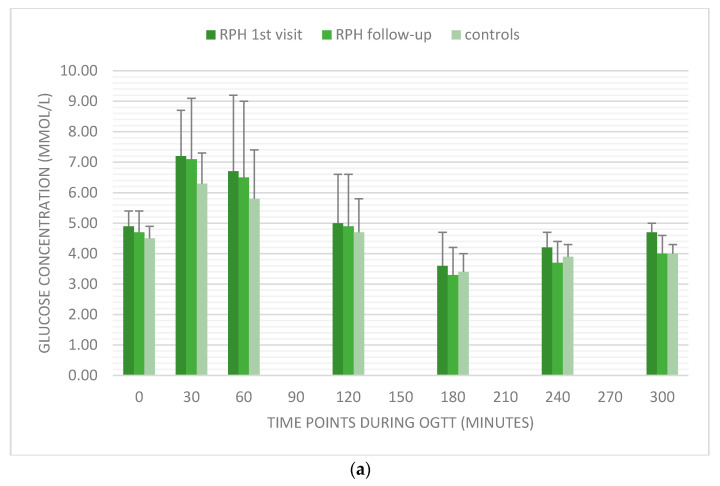

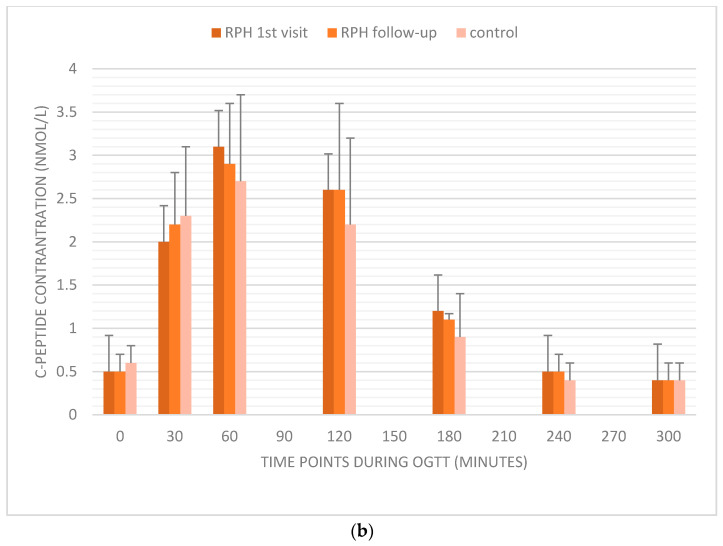
(**a**). Comparison of blood glucose concentration in subjects with RPH at baseline and follow-up vs. the healthy control group, showing no difference in any time point during 5-h OGTT. (**b**). Comparison of C-peptide concentration in subjects with RPH at baseline and follow-up vs. the healthy control group, showing no difference in any time point during 5-h OGTT. Legend: RPH: reactive postprandial hypoglycemia, OGTT: oral glucose tolerance test.

The control group did not differ in glucose, insulin, and c-peptide concentrations from the RPH group during the OGTTs (Figure 1a,b, Supplementary Figure S1).

In RPH individuals, the test was positive for hypoglycemia occurrence in 48% of cases. In the majority (69%) of individuals, hypoglycemia occurred in the 180th minute of the 5-h OGTTs, and in 31% of individuals, it occurred in the 120th minute of the 5-h OGTTs. The individuals in the control group developed hypoglycemia in 90% of cases during the OGTTs, and of those, 90% experienced it in the 180th minute.

## 4. Discussion

In this pilot study, we re-evaluated the glycemic response in non-diabetic women experiencing postprandial symptoms attributed to hypoglycemia, followed by a case-control study. We found no biochemical deterioration of beta-cell secretory capacity and no progression to dysglycemia after, on average, 6.4 ± 4.2 years of follow-up. Compared to healthy control subjects, individuals with postprandial hypoglycemia exhibited no differences in concentrations or in the shape of the glucose or insulin curve during the 5-h OGTTs. The subjects with RPH were not insulin resistant, and their insulin sensitivity did not deteriorate during the observation period. 

To examine the predictive value of RPH occurrence on progression to dysglycemia, we evaluated paired glucose curves and surrogate indices of insulin sensitivity or beta-cell failure by prospectively documenting biochemical characteristics with 5-h OGTTs in 29 female subjects. The subjects were non-obese; their HbA1c was lower at follow up, and no significant intra-individual differences in the indices of insulin sensitivity or resistance were noted during the observation period. Glucose concentrations were even lower at the follow-up visit at certain postprandial time points. According to HOMA-IR and the Matsuda index, subjects with RPH were not insulin resistant but categorized as insulin sensitive. Identifying hyperinsulinemia in the absence of glucose intolerance is becoming increasingly important in predicting future diabetes risk [21]. Many studies suggest that RPH is a hallmark of insulin resistance, since supposedly obese people have higher rates of reactive hypoglycemia than other groups [22]. This notion is supported by previous publications of increased prevalence of RPH in women with insulin-resistant conditions, e.g., polycystic ovary syndrome [23]. On the other hand, increased insulin sensitivity has been proven in RPH subjects by many authors [14,24,25], which is more in line with our results. Furthermore, some recent publications confidently state that no metabolic derangements are found in subjects with RPH [5] and that in patients reporting hypoglycemia, glucoregulatory hormone responses are similar to those in the controls [26]. Therefore, we further age- and BMI-matched the RPH group of subjects with the control group. Compared to the healthy control subjects, individuals with postprandial hypoglycemia showed no differences in glucose or insulin secretion upon stimulation.

The insulinogenic index (IGI) represents the first-phase insulin response, calculated based on the changes in insulin and glucose over the first 30 minutes after glucose load in the oral glucose tolerance test. The insulinogenic index and its composite with insulin sensitivity (disposition index) have been demonstrated to be predictive of type 2 diabetes. The disposition index correlates strongly with glucose tolerance, such that individuals with the lowest disposition index are at increased risk of type 2 diabetes [18,19]. The debate on the interaction and relative importance of insulin resistance or impaired beta-cell function as the primary event in the pathogenesis of type 2 diabetes continues [27,28]. Our results indicate that subjects with RPH had insulin secretion comparable to that in healthy controls; however, the insulinogenic index, indicating the first-phase insulin response, was significantly lower, though still above the proposed threshold for insufficient insulin secretion. This finding could be interpreted in two ways. In the healthy control group, a better first-phase insulin secretion response to glucose load could have been aimed at compensating their borderline insulin resistance (HOMA of 2.5), or alternatively, subjects with RPH had a blunted first-phase insulin response as a primary defect in glucose regulation. On the other hand, blunted insulin secretion could have been an adaptive response to high insulin sensitivity in our study subjects, since according to some authors, beta-cell function varies quantitatively with differences in insulin sensitivity, and the initial increase in plasma glucose is determined primarily by hepatic insulin resistance [29,30]. According to the literature, beta-cell function in RPH is preserved, and even higher indices of insulin secretion have been described [24]. It should also be taken into account that only one index of insulin secretion was significantly different between the groups in our cohort, which could also have been a consequence of intra-individual variation in insulin concentrations, since it has been established that random test–retest differences in insulin concentrations are important (e.g., 61% test–retest difference in fasting insulin and 125% in 120th minute insulin), especially in small groups of individuals [31]. 

The plasma glucose shape during an OGTT depends on glucose tolerance and could be used as a metabolic screening tool [32], since the glucose concentrations measured in the fasting state (0. min), as well as intermediate measures (30th and 60th minutes), during an OGTT may provide additional information regarding a person’s risk of future type 2 diabetes and even mortality [33,34,35,36]. In the RPH group, the glucose response showed a favorable pattern [33], with a peak (average) glucose concentration at the 30th minute below 8 mmol/L. Our cohort average 60th-minute-glucose concentration was 6.7 ± 2.5 during the first visit and 6.5 ± 2.5 mmol/L during the follow-up visit. In the Botnia study and Malmö Prevention Project, 60th-minute-glucose concentrations of 8.9 mmol/L and 8.4 mmol/L were the optimal cut-off points for the initial screening and selection of high-risk individuals, respectively. In these two cohorts, high-risk individuals had a substantially increased risk of incident type 2 diabetes (ORs of 8.0 and 3.8) and captured 75% and 62% of all incident type 2 diabetes [37]. Regarding insulin secretion patterns, no unfavorable responses in RPH subjects were documented either. The RPH subjects peaked in insulin concentration 60 min after stimulation, which is known to be more favorable than late peaks (e.g., 120th-minute peaks), which predict higher future type 2 diabetes risk in individuals with normal glucose tolerance [36,38]. 

Unexpectedly, we found a remarkably high occurrence of biochemical hypoglycemia during the OGTTs in healthy controls, as well as poor reproducibility of hypoglycemia in the paired OGTTs in test subjects. This finding is in concordance with previous publications that indicate the overall reproducibility of OGTTs to be between 65% and 75% in subjects with normal glucose tolerance, impaired glucose tolerance (IGT), or diabetes [39,40]. Many factors are known to have an impact on OGTT results, from pre-test, pre-analytical, and analytical to postanalytical factors [40,41]; therefore, its validity is partly questionable, which was also evident in our study. A contentious issue remaining to be resolved is whether postprandial symptoms are actually related to low blood glucose [42,43] or any metabolic disturbances [5,26,44]. The occurrence of biochemical hypoglycemia was as high as 90% in healthy controls, yet it was only triggered in 48% of subjects known to be experiencing hypoglycemia symptoms, indicating a possibility of pseudohypoglycemia, as assessed by Simpson et al. using continuous glucose monitoring [43]. This finding is partly in line with frequent chemical hypoglycemia occurrence in healthy controls (25%) in other research settings [26]. On the other hand, despite evidently lacking specificity and reproducibility, the OGTT remains a suitable tool to formally recreate the circumstances in which symptomatic hypoglycemia occurs, as suggested by the Endocrine Society recommendations [1]. Despite different authors raising concerns about the reproducibility of the OGTT for over 50 years [39,45,46], it remains the current “gold standard” for diagnosing type 2 diabetes.

This study had many limitations that are intrinsic to a pilot study, mostly consisting of a small sample size and consequently a limited number of measurements. In terms of assessing the shape of the glucose curve, we lacked the 90th-minute-point concentrations, which could have allowed us to further speculate on metabolic processes, since some authors distinguish the biphasic and monophasic forms of glucose curves during OGTTs [32]. The surrogate indices of insulin secretion and insulin sensitivity calculated in our study were not gold-standard clamp-derived indices [17]; however, we believe that HOMA-IR and the Matsuda index are widely used [47]. Possible ethnic differences were not included either, since we included exclusively the Caucasian population, which is prevalent in our country. We further lacked data on possible inter-individual differences in gastric emptying during the OGTTs, which have been described as a determinant of the glycemic response, most notably of the initial increase (30th minute) [48]. We also acknowledge that altered glucose responses to the OGTT are possible during different phases of the menstrual cycle, but unfortunately we did not perform all the OGTTs in the same phase of the menstrual cycle [49,50]. Furthermore, premenopausal women exhibit enhanced insulin sensitivity, but this advantage disappears after menopause, owing to a reduction in circulating 17β-estradiol [51]. However, since the participants were premenopausal at the initial visit and still at follow-up, we did not expect declines in estrogen concentration. However, menstrual cycle-related fluctuations in estrogen levels could still affect insulin and glycemic response to glucose load.

One of the strengths of the present study was the selection of a population without associated causes of hypoglycemia, carefully excluding any cases of known endocrinopathy and even of IFG or IGT, as well as patients with a personal history of bariatric surgery, which is known to predispose patients to postprandial hypoglycemia [52]. We believe that by including only female subjects, a greater homogeneity of the group was ensured. Females predominated among people with RPH in the entire initially referred cohort (80%), as well as in similar studies published in this context [5,6,7,43]. We believe that the use of the prolonged, 300-min (5-h) OGTT is far more suitable than the standard 120-min OGTT in this clinical setting, since reactive hypoglycemia occurs after the 120th minute and can be, in this manner, properly documented. In line with this, guidelines of the Endocrine Society [1] explicitly advise using a 5-h test instead of shorter ones to accurately diagnose RPH. Nevertheless, guidelines advise the use of a mixed-meal test instead of the OGTT; however, they admit at the same time that there exist no standards for the interpretation of the mixed-meal test [1]. The 75 g OGTT and the mixed-meal test have been widely criticized in this setting [1,2,26], since the 5-h OGTT has been firmly established as one of the standard diagnostic tests in non-diabetic hypoglycemia at our clinic, we chose to use the same test to be able to compare current and past results. In addition, the outline of a 5-h glucose or insulin profile following stimulation is rarely described in the literature and could offer new insights into hormonal dynamics [5,22,53,54]. To our knowledge, this is the first prospective study using the 5-h OGTT in an RPH-experiencing population.

Further research should focus on the clinical definition of RPH, defining the proposed method for identification and a standardized cut-off glucose value. This could assist in more precise prevalence estimations and health implications, especially the predictive value for incident type 2 diabetes development. 

In conclusion, we found that subjects with RPH proved to be insulin sensitive and demonstrated no biochemical deterioration of beta-cell secretory function or new-onset type 2 diabetes during the observation period. We found no indices of increased incident type 2 diabetes risk, except for a blunted first-phase insulin response compared with age- and BMI-matched controls that did not exceed the reference range. This dictates the need for further research to confirm the hypothesis of future diabetes risk in individuals experiencing RPH. Furthermore, our research adds to the body of evidence questioning the use of OGTTs in diagnosing RPH due to low specificity and reproducibility.

## Figures and Tables

**Table 1:** Anthropometric and biochemical characteristics of subjects with RPH (*n* = 29) at baseline and at the follow-up visit after on average 6.4 ± 4.2 years.

	Baseline Visit	Follow-Up Visit	
Variable Value			*p*-Value
Body weight (kg)	67.6 ± 11.3	69.3 ± 10.11	0.141
BMI (kg/m^2^)	23.7 (20.4, 26.2)	24.5 (21.3, 26.8)	0.215
HbA1c (%/ mmol/mol))	5.3 (4.9, 5.6) * **/34.4 (30.1, 41.0)**	5.0 (4.8, 5.3) * **/31.8 (29.0, 34.4)**	**0.004**
Matsuda index	9.3 (5.3, 12.5)	9.2 (3.6, 57.4)	0.620
HOMA -IR	1.07 ± 0.74	1.08 ± 1.2	0.985
Insulinogenic index	0.84 (0.59, 1.7)	0.73 (0.43, 1.5)	0.069
Disposition index	7.4 (5.1, 19.4)	5.4 (2.0, 29.7)	0.283

Means ± standard deviations or medians (25th, 75th percentile) are presented as appropriate. Statistically significant values are presented in bold with an asterisk. Legend: BMI, body mass index; HOMA-IR, Homeostatic Model Assessment for Insulin Resistance.

**Table 2:** Comparison of relevant anthropometric and biochemical characteristics of subjects in the RPH group vs. the control group.

	RPH Group	Control Group	*p*-Value
Age (years)	37.2 ± 10.0	39.7 ± 9.2	0.515
Body weight (kg)	68.6 ± 10.0	70.3 ± 11.1	0.640
Waist circumference (cm)	84.8 ± 12.0	84.2 ± 10.6	0.886
Hip circumference (cm)	102.8 ± 8.6	104.1 ± 7.9	0.755
WHR	0.8 ± 0.1	0.8 ± 0.1	0.675
BMI (kg/m^2^)	24.6 ± 3.5	24.8 ± 4.3	0.877
Systolic blood pressure (mmHg)	107 ± 10	108 ± 9	0.884
Diastolic blood pressure (mmHg)	70 ± 7	72 ± 8	0.618
Heart rate (beats/min)	61 ± 10	64 ± 11	0.423
LDL-c (mmol/L)	2.9 ± 0.61	2.66 ± 0.56	0.546
TG (mmol/L)	1.1 ± 0.41	0.84 ± 0.33	0.078
HbA1c (%; mmol/mol)	5.0 ± 0.3 31.1 ± 2.1	5.2 ± 0.6 33.3 ± 4.2	0.593
Matsuda index	9.2 (3.6, 57.4)	6.0 (4.0, 76.0)	0.804
HOMA-IR	1.08 ± 1.2	2.5 ± 4.2	0.136
Insulinogenic index	0.73 (0.43, 1.5) *	1.0 (1.0, 3.0) *	0.028 *
Disposition index	5.4 (2.0, 29.7)	16.0 (7.0, 42.0)	0.217

Means ± standard deviations or medians (25th, 75th percentile) are presented as appropriate. Statistically significant values are presented in bold with an asterisk. TG, serum triglycerides concentration. Legend: WHR, waist-to-hip ratio; BMI, body mass index; HOMA-IR, Homeostatic Model Assessment for Insulin Resistance; LDL-C, cholesterol; TG, triglycerides.

## Data Availability

The data presented in this study are available in article and Supplementary Material.

## Supplementary Materials

The following supporting information can be downloaded at: https://www.mdpi.com/article/10.3390/metabo12121232/s1. Supplementary Table S1. Pharmacotherapy of comorbidities in RPH participants (*n* = 29). Supplementary Table S2. Blood glucose, insulin, and C-peptide concentration during 5-h OGTT in individuals with RPH at baseline and at follow-up. Supplementary Figure S1. Comparison of insulin concentration in subjects with RPH at baseline and follow-up vs. the healthy control group, showing no difference in any time point during 5-h OGTT.
