# Supplementary material for: No Indices of Increased Type 2 Diabetes Risk in Individuals with Reactive Postprandial Hypoglycemia
Source: Metabolites. 2022 Dec 8;12(12):1232. doi: 10.3390/metabo12121232 (PMC9787184; doi:10.3390/metabo12121232)
Supplement: Supplementary file 1 [file metabolites-12-01232-s001.zip › metabolites-2054941-supplementary.pdf]

Supplementary Table S1.: Pharmacotherapy of comorbidities in RPH participants (n = 29).

Class of medication	*n* (%)
Proton pump inhibitors	5 (17)
Antidepressant/antipsychotics	5 (17)
Thyroid substitution (levothyroxine)	4 (13)
Bronchodilatator	2 (7)
ACE inhibitor/calcium channel blocker	2 (7%)
Statin	2 (7%)
Beta receptor blocker	1 (3%)
Methotrexate	1 (3%)

LEGEND: ACE, angiotensin-converting enzyme.

Supplementary Table S2. Blood glucose, insulin, and C-peptide concentration during 5-h OGTT in individuals with RPH at baseline and at follow-up.

Time (min)	Glucose (mmol/L)			Insulin (mU/L)			C-peptide (nmol/L)		
	**1. Visit**	**Follow Up**	***p*-Value**	1. Visit	Follow Up	*p*-Value	1. Visit	Follow Up	*p*-Value
**0**	4.9 ± 0.5	4.7 ± 0.7	0.121	4.5 ± 3.2	4.4 ± 4.2	0.977	0.5 ± 0.1	0.5 ± 0.2	0.756
**30**	7.2 ± 1.5	7.1 ± 2.0	0.822	39.7 ± 22.9	43.0 ± 17.6	0.614	2.0 ± 0.7	2.2 ± 0.6	0.262
**60**	6.7 ± 2.5	6.5 ± 2.5	0.581	64.6 ± 30.5	52.6 ± 18.5	0.149	3.1 ± 0.9	2.9 ± 0.7	0.312
**120**	5.0 ± 1.6	4.9 ± 1.7	0.847	47.4 ± 49.0	43.7 ± 26.0	0.743	2.6 ± 1.3	2.6 ± 1.0	0.978
**180**	3.6 ± 1.1	3.3 ± 0.9	0.174	14.8 ± 14.0	13.5 ± 11.2	0.633	1.2 ± 0.8	1.1 ± 0.7	0.364
**240**	**4.2 ± 0.5***	**3.7 ± 0.7***	**0.007***	5.5 ± 5.5	5.8 ± 5.1	0.833	0.5 ± 0.3	0.5 ± 0.2	0.841
**300**	**4.7 ± 0.3***	**4.0 ± 0.6***	**0.000***	4.2 ± 4.0	3.7 ± 3.0	0.682	0.4 ± 0.2	0.4 ± 0.2	0.904

Means ± standard deviations are presented. Statistically significant values are presented in bold with an asterisk.

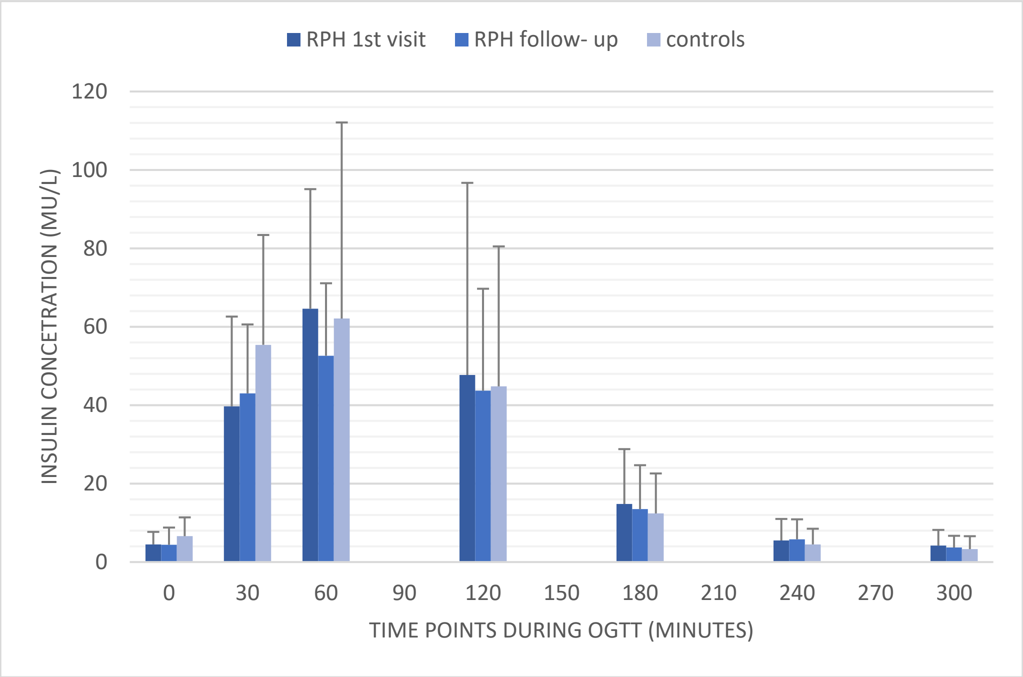

Supplementary Figure S1. Comparison of insulin concentration in subjects with RPH at baseline and follow-up vs. the healthy control group, showing no difference in any time point during 5-h OGTT.

Legend: RPH: reactive postprandial hypoglycemia, OGTT: oral glucose tolerance test.
